# Induction of Chromosomal Instability via Telomere Dysfunction and Epigenetic Alterations in Myeloid Neoplasia

**DOI:** 10.3390/cancers5030857

**Published:** 2013-07-04

**Authors:** Beate Vajen, Kathrin Thomay, Brigitte Schlegelberger

**Affiliations:** Institute of Cell and Molecular Pathology, Hannover Medical School, Carl-Neuberg-Str. 1, 30625 Hannover, Germany; E-Mails: vajen.beate@mh-hannover.de (B.V.); thomay.kathrin@mh-hannover.de (K.T.)

**Keywords:** chromosomal instability, myeloid leukemia, MDS, AML, telomeres, epigenetics, genetic instability

## Abstract

Chromosomal instability (CIN) is a characteristic feature of cancer. In this review, we concentrate on mechanisms leading to CIN in myeloid neoplasia, *i.e*., myelodysplastic syndrome (MDS) or acute myeloid leukemia (AML). The pathogenesis of myeloid neoplasia is complex and involves genetic and epigenetic alterations. Chromosome aberrations define specific subgroups and guide clinical decisions. Genomic instability may play an essential role in leukemogenesis by promoting the accumulation of genetic lesions responsible for clonal evolution. Indeed, disease progression is often driven by clonal evolution into complex karyotypes. Earlier studies have shown an association between telomere shortening and advanced MDS and underlined the important role of dysfunctional telomeres in the development of genetic instability and cancer. Several studies link chromosome rearrangements and aberrant DNA and histone methylation. Genes implicated in epigenetic control, like *DNMT3A*, *ASXL1*, *EZH2* and *TET2*, have been discovered to be mutated in MDS. Moreover, gene-specific hypermethylation correlates highly significantly with the risk score according to the International Prognostic Scoring System. In AML, methylation profiling also revealed clustering dependent on the genetic status. Clearly, genetic instability and clonal evolution are driving forces for leukemic transformation. Understanding the mechanisms inducing CIN will be important for prevention and for novel approaches towards therapeutic interventions.

## 1. Introduction

### 1.1. Chromosomal Instability in Cancer

Maintenance of genomic integrity is of the utmost importance for an organism’s survival and a prerequisite for successfully passing on genetic information. However, the stability of the genome is constantly threatened by DNA replication errors and by DNA damage due to a multitude of exogenous and endogenous factors, e.g., oxidative stress. The cell continuously counteracts these processes and has developed a complex network to repair genetic alterations. Nonetheless, a small number of cells may not succeed in repairing damaged DNA. In this situation, the cell usually enters apoptosis. However, in some cases mutations, like in oncogenes or tumor suppressor genes, may occur that allow the cell to bypass apoptosis and to survive. If such a cell acquires additional genetic alterations providing a clonal advantage, this may lead to malignant transformation and ultimately to cancer [1].

Genomic instability may result in numerous defects like replication errors, telomere dysfunction, epigenetic changes or defective DNA repair as a few examples. Chromosomal instability (CIN), a form of genomic instability, is by definition the cell-to-cell variability with regard to chromosomal changes, e.g., the rate of gross chromosome changes like an imbalance in the number of chromosomes per cell and an enhanced rate of loss of heterozygosity [2,3,4]. As such, chromosomal instability may predispose to the outgrowth of clones containing “fixed” chromosome aberrations. Chromosome aberrations in myeloid neoplasms include whole-chromosome aberrations like monosomy or trisomy or structural changes like deletions, translocations and inversions [5]. Therefore, Bajani *et al*. [6] suggest to distinguish between structural chromosomal instability (S-CIN), developed at unstable genomic regions, or through aberrant DNA repair or methylation and numerical CIN (N-CIN), which occur due to mitotic segregation errors. Chromosomal instability can be found in all kinds of human cancer and is usually a strong negative prognostic indicator [7,8]. Also in myelodysplastic syndromes, elevated chromosomal instability, *i.e*., the cell-to-cell variability as determined by fluorescence *in situ* hybridization, correlates with poor outcome, irrespective of the cytogenetic subtype [9].

### 1.2. Role of Chromosomal Aberrations in Human Leukemia

In this review we discuss the role of chromosomal and genomic instability in myeloid neoplasia, like myelodysplastic syndrome (MDS) and acute myeloid leukemia (AML). MDS are a heterogeneous group of malignant clonal disorders of hematopoietic stem cells, characterized by dysplastic cells and an ineffective hematopoiesis in one or more myeloid cell lineages [10]. According to the World Health Organization (WHO), MDS is subdivided into different morphological subtypes. The International Prognostic Scoring System (IPSS) categorizes these subtypes into different risk groups (low, intermediate 1 and 2 and high) based on the number of cytopenias, the karyotype, and the percentage of blasts in the bone marrow [10,11]. Generally, in MDS, the amount of blasts, the immature hematopoietic progenitor cells, in the bone marrow is less than 20%. This definition distinguishes it from AML with an amount of blasts of more than 20% [12]. In about 10% to 50% of cases, strongly depending on the respective subtype, myelodysplastic syndromes show a propensity to progression into AML [13,14].

In MDS, clonal chromosome aberrations have important diagnostic, prognostic and clinical relevance [10,15]. In a high amount of cases, MDS shows progression into AML, which is often associated with karyotypic evolution, *i.e*., the acquisition of additional chromosomal aberrations.

In general, clonal chromosome aberrations can be found in about half of the AML and MDS patients [16]. MDS shows mainly unbalanced aberrations [17]. The most common chromosomal aberrations in MDS are monosomy 7, loss of the Y chromosome, trisomies 8 and 21 and deletions in 5q, 7q, 17p and 20q. Losses of 5q, 7q and 17p are also frequent aberrations of complex karyotypes, which by definition contain three or more clonal aberrations.

Cytogenetic abnormalities in AML and MDS are associated with distinct clinical and morphological subtypes and often predict disease outcome. For example, MDS with an isolated del(5q) defines a subtype of MDS with a favorable outcome, a median survival of 77.2 months, and a low risk of transformation into AML. In contrast, complex karyotypes are associated with a very high risk of transformation into AML and with a very poor prognosis, *i.e*., a median survival of 8.8 months [10]. Since complex karyotypes frequently occur in patients with secondary MDS, who have been treated with combined chemo- and radiotherapy due to a primary malignancy, increased genomic instability may lead to the development of aberrant clones.

As MDS typically shows unbalanced aberrations like deletions and monosomies, it can be concluded that the molecular mechanism in MDS is predominantly loss or inactivation of a tumor suppressor gene, in contrast to AML, where often balanced translocations and inversions occur that may induce proliferation or inhibit differentiation [16,18]. As examples, a translocation t(8;21)/*AML1-ETO* fusion and an inv(16)/*CBFb-MYH11* fusion result in the inactivation of core binding factors that have an essential role in myeloid differentiation. Both AML subtypes are associated with a good prognosis [12]. A t(15;17)/*PML-RARA* fusion resulting in the inactivation of the retinoid acid receptor α also defines an AML subtype with good prognosis. The molecular defect can be counteracted by treatment with retinoic acid, which induces the differentiation of the myeloid blasts [19]. Inversion of 3q, translocations of *MLL*, a t(6;9)/*DEK-NUP214* fusion or a complex aberrant karyotype are each associated with a poor prognosis [12,20,21].

Genes frequently found to be mutated in karyotypically normal AML are *NPM1*, *FLT-3* and *CEBPalpha* [22]. Recently, recurrent mutations with prognostic significance have been discovered in MDS, among those mutations in the genes *TP53*, *EZH2*, *ETV6*, *GNAS*, *RUNX1*, and *ASXL1* [23].

### 1.3. Mechanisms to Induce CIN in Myeloid Neoplasia

The pathogenesis of myeloid neoplasia is generally complex and involves genetic, epigenetic and immune-mediated mechanisms [11]. As in many cancer types, chromosomal instability and clonal evolution play an essential role in leukemogenesis by promoting the accumulation of genetic lesions responsible for malignant transformation. Although exogenous factors like previous chemo- and radiotherapy increase the risk to develop MDS and AML, and particularly those with complex karyotypes, the exact mechanisms inducing CIN are not clear.

Aberrations specific for MDS and AML, particularly terminal deletions, unbalanced translocations as well as gains and losses, could theoretically result from dysfunctional telomeres [24]. Earlier studies have shown an association between shorter telomeres and advanced MDS [25,26]. Also in AML, an increasing number of aberrations seem to be associated with ongoing telomere erosion, resulting in critically short telomeres of one or more chromosomes [24]. Data from animal models and *in vitro* experiments have elucidated an important role of dysfunctional telomeres in the development of genetic instability and cancer, particularly MDS and AML [24,27]. Recent studies not only underlined the significance of the mean telomere length, but particularly of single or very few critically short, dysfunctional telomeres causing chromosomal instability [28,29]. In the clinical context, however, the relevance of critically short telomeres for the development of CIN in MDS and AML remains to be shown.

The identification of recurrent mutations in genes involved in the epigenetic regulation in patients with MDS has led to new insights into the pathophysiology of this disorder. Of particular interest is the recent recognition of mutations in genes involved in histone modification (*EZH2* and *ASXL1*) and DNA methylation (*DNMT3A* and *TET2*). For time-controlled activation and silencing of tissue-specific genes in eukaryotic cells, continuous remodeling of the chromatin structure is necessary. Epigenetic factors modify the DNA and DNA-associated histones, thereby inducing conformational changes of the chromatin that allow activation or repression of gene expression. Mutations of epigenetic modifiers provide an important link between genetic and epigenetic alterations in MDS. In AML, gene expression studies also observed differential expression of the *DNMT3A* and *DNMT3B* genes, coding for DNA methyltransferases [30]. Furthermore, aberrant DNA methylation patterns helped to identify new subgroups of AML. Notably, cytogenetic subgroups are characterized by distinct DNA methylation patterns. Particularly for AML with normal karyotype, they define a methylation-based outcome predictor for disease-free and overall survival [31,32].

Recently, the important role of histone methylation for the induction of chromosomal and genomic instability and leukemia pathology in humans and mice became evident. Of course, it is not only telomeric dysfunction or epigenetic alterations that play an important role in the induction of chromosomal and genomic instability in myeloid malignancies. Chromosome aberrations have, for example, also been described to occur due to defective cytokinesis or cell cycle regulation [33,34]. Also, increased DNA damage, either via internal mechanisms leading to increased reactive oxygen species (ROS) or external factors like chemicals, cancer therapy or radiation, or a defective DNA damage repair in general due to mutations, may lead to aberrations in myeloid leukemia [35,36,37].

Yet, the mechanisms by which epigenetic alterations and telomere dysfunction contribute to disease pathogenesis are a highly active area of research and therefore we would like to address the following question in this review: How can telomeres and epigenetics contribute to inducing chromosomal and genomic instability and what role does this play in leukemogenesis?

## 2. Telomeres

### 2.1. Telomeres and CIN

Progressive telomere shortening due to the end-replication problem is one of the molecular mechanisms underlying aging. Starting at a length of around 15 kb and with an approximate telomere loss of 30–150 bp with each cell division, continued telomere length shortening can be observed over time. However, within the first two years of life, a more rapid loss of 1,000–3,000 bp/year takes place. Furthermore, accelerated telomere shortening can also be observed after the age of 60 years. The reason for this might, for example, lie in replicative stress due to a diminishing hematopoietic stem cell pool [38]. Yet, excessive telomere shortening also occurs during excessive proliferation after stem cell depletion or in cancer cells.

As already mentioned, short telomeres, e.g., due to excessive proliferation, induce senescence when they reach a critical length of about 5–8 kb, the so-called Hayflick limit [39]. Importantly, single critically short telomeres are sufficient to elicit this cellular response. This finding demonstrates that, besides the median telomere length of the cell, the length of the single chromosome arms is also of great importance [40].

Dysfunctional telomeres lead to senescence and thus limit the proliferative potential of a stem cell. This might possibly lead to selection of stem cells with a defective DNA damage response, in which no cell-cycle arrest occurs due to deficient or low repair capacity and which are therefore prone to chromosomal instability predisposing them for leukemia [41].

Usually, cells that are able to circumvent senescence enter a further growth arrest phase (crisis), in which critically short telomeres of about 3–4 kb tend to fuse with other “free ends” and thus promote further genomic instability [42]. Elimination of cells with short telomeres due to either aging or excessive proliferation is thus an important tumor-suppressive mechanism of the cell and a hurdle to tumor progression [43,44].

**Figure 1:**
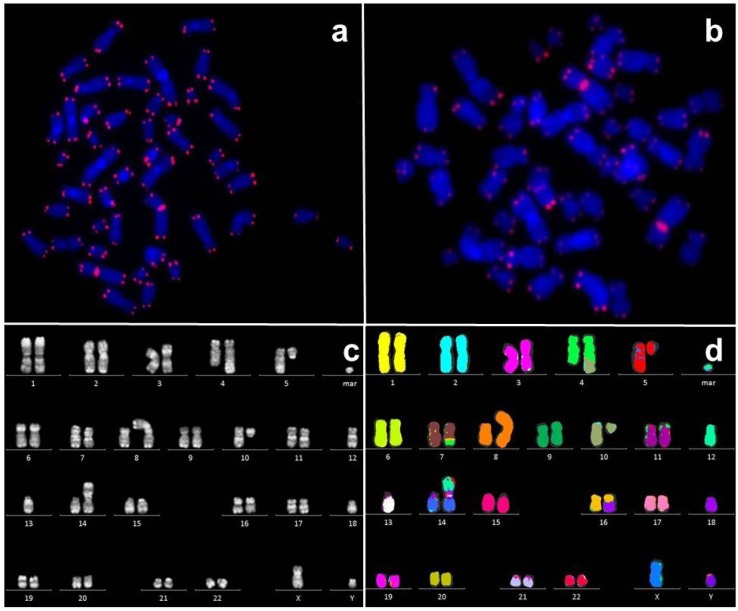
Imaging methods for chromosomal instability and shortened telomeres (**a**) Telomere/Centromere (T/C)-FISH metaphase of a healthy individual; (**b**) T/C-FISH metaphase of a patient with MDS and shortened telomeres; (**c**) Fluorescence R-banding karyotype of a patient with AML and complex aberrant karyotype, (**d**) Multicolor FISH karyotype of the same patient.

However, in the case of further mutations as in *TP53* and *RB*, cells may be able to bypass senescence and crisis. In the case of continued proliferation, the cells with short telomeres might turn into immortalized cancer stem cells, in which chromosomal aberrations are stabilized by up-regulation of telomerase or by induction of ALT [45,46] (Figure 1). In line with this, in MDS a complex karyotype is observed more often in older patients, naturally with a background of shorter telomeres, than in younger patients, pointing towards short telomeres as a risk factor for chromosomal instability.

Cancer stem cells generally show genetic aberrations, telomere stability and telomerase activity, suggesting that these cells have initially gone through a phase with very short telomeres, which have then at a later stage been re-elongated and stabilized [42,47].The appearance of these telomerase-positive clones with stabilized chromosome aberrations might have evolved due to the selective growth advantage of cells with activated telomerase [44].

Thus, critically short telomeres, even single short telomeres within a cell, can induce end-to-end fusions and the formation of chromatin bridges during anaphase with subsequent breakage-fusion-bridge events (B/F/B cycles). These events can lead to either structural rearrangements due to sister chromatid fusions, extensive chromatin fragmentation, to the loss of whole chromosomes via mechanical disruption of the spindle machinery or to failure of cytokinesis leading to polyploidization and multiple spindle configurations [48,49]. Cycles of breakage-fusion-bridge events stop after acquisition of new telomeres by non-reciprocal translocation to the end of the unstable chromosome (telomere capture).

### 2.2. Telomeres in Leukemia

During the development of MDS and AML, due to excessive proliferation and/or another underlying defect, pathologically shortened telomeres in a hematopoietic stem or progenitor cell may eventually reach a critically short length. As described, this may lead to increased CIN and genetic aberrations [48]. Aberrations typical for these diseases, particularly terminal deletions, unbalanced translocations as well as gains and losses of whole chromosomes could theoretically result from dysfunctional telomeres [24].

Specifically, short telomeres may lead to the generation of dicentric chromosomes, which may induce B/F/B cycles that may subsequently lead to loss or gain of chromosomes or to the development of unbalanced changes, typical for MDS. If these chromosome aberrations provide a survival benefit or a proliferative advantage, this may then, at a later stage, lead to clonal evolution of aberrant clones and possibly to the re-activation of telomerase. Indeed, increased telomerase activity has been described in patients with advanced forms of MDS [50]. Also in AML, increased TERT expression, usually a characteristic feature of cancer cells, can be found in correlation with the complexity of the karyotype as well as the disease severity [51].

An association with MDS, especially with regard to the severity of the subtype and short telomeres, has already been shown [25,26,52]. As shown by Boultwood *et al*., telomere length showed a certain degree of heterogeneity within the subtypes but short telomeres could still be linked to leukemic transformation and complex aberrant karyotypes. Also, Sieglova *et al*. showed an association between telomere shortening and disease progression suggesting telomere dynamics as a prognostic factor in MDS. Likewise, an increasing number of aberrations in AML has been described to be associated with ongoing telomere erosion on the one hand and critically short telomeres on the other, pointing towards an essential role of telomeres in the pathogenesis of these diseases [24,53].

Recent studies not only underlined the significance of the mean telomere length, but in particular of single or very few critically short, dysfunctional telomeres causing CIN [28,29]. As an example, it has already been shown in mTR^−/−^mice that chromosomes frequently involved in aberrations have the shortest telomeres, leading to the conclusion that these short dysfunctional telomeres limit cellular survival [28].

Studies profiling the three-dimensional architecture of telomeres in patients with MDS and AML have shown that, based upon parameters like telomere numbers, telomere aggregates, signal intensities, nuclear volumes and nuclear telomere distribution, patients can be subdivided into distinct subgroups [54]. Notably, the evolution of telomeric dysfunction is linked to the progression of MDS into AML.

The role of telomeres, especially in MDS, becomes clear when considering diseases like dyskeratosis congenita (DC) or aplastic anemia (AA), in which a mutation in one of the telomerase components or of the “shelterin” complex (telomere-binding and stabilizing proteins) leads to critically short telomeres (<5 kb) and a substantially increased risk of MDS [55]. DC is a premature aging syndrome that—among other symptoms—is characterized by cytopenia in one or more lineages. This is why DC is often first diagnosed when MDS develops. Besides loss-of-function mutations in one of the telomerase components, mainly *TERT*, *TERC* and *DKC*, but also *NOP10* and *NHP2*, mutations in the telomere-associated protein TINF2 have also been found in patients with DC [56,57]. These mutations lead to either telomerase deficiency (as with the X-linked *DKC1*), a dysfunctional telomere repair complex (as with the autosomal dominant *TERT* and *TERC* mutations) or disrupted telomeres (as with the *TINF2* mutations). Although showing differences in the clinical manifestation as well as the severity of symptoms, all these mutations lead to shortened telomeres [56].

Recently, hypomorphic mutations of *TERT* have been found in a subset of patients with AML that also seemed to be associated with the occurrence of specific aberrations, in particular trisomy 8, inversion 16, a translocation t(15;17) and a complex aberrant karyotype [41]. In aplastic anemia and MDS, somatic mutations in *TERT* or *TERC* can be observed [58]. In AA, short telomeres have directly been linked to the probability of developing a cytogenetically abnormal clone, underlining the role of telomerase and short telomeres in malignant transformation of hematopoietic stem cells and MDS development [58,59,60].

Intriguingly, these data provide a first hint that short telomeres contribute to the pathogenesis of MDS and AML. However, the extent to which short telomeres induce chromosomal instability and the development of aberrant clones still remains to be elucidated. It is also of high importance for patient management, whether the current telomere length may predict the risk to develop MDS and AML and whether a patient’s telomere length is able to predict the risk of progression and relapse.

### 2.3. Telomere Length Measurement in Primary Cells of Patients and Our Data

According to our hypothesis, different cytogenetic subtypes of MDS may show different telomere length profiles. Possibly those chromosomes involved in or prone to specific aberrations may have shorter telomeres than normal chromosomes. These critically short telomeres may be a driving force towards leukemic transformation and clonal evolution. However, they might be masked in the average telomere length by longer telomeres of other chromosomes in the same cell. Therefore, determining the telomere length of each chromosome is necessary to answer this question.

To elucidate this, we recently selected a cohort of 78 patients with different cytogenetic and morphological subtypes of MDS and a cohort of 18 age and gender-matched healthy controls for telomere length measurement. In particular, we investigated the telomere length profiles and the role of single critically short telomeres by applying telomere/centromere-FISH (T/C-FISH) [61]. This method, basically a quantitative FISH in which the telomeric signal intensities are set in relation to the signal intensity of centromere 2 as an internal reference, was used in combination with fluorescence R-banding analysis of the same metaphases. This particularly allowed telomere length measurement of each single distinct chromosome arm and importantly, in the case of patients with an aberrant karyotype, guaranteed measurement of the aberrant clone excluding falsification of the results by normal cells. Bone marrow cells of patients with MDS showed significantly shorter telomeres than those of healthy controls. However, no association between short telomeres and specific cytogenetic or morphological subtypes was found and telomere lengths did not differ significantly between distinct morphological subtypes of MDS. Yet, so-called neo-telomeres (telomeres at the fusion site of two chromosomes) were found in two patients with a complex karyotype, showing a role of short telomeres in disease pathogenesis. We therefore hypothesized that, in most patients with MDS and monosomy 7 or complex karyotype, formerly short telomeres are stabilized or elongated by reactivation of telomerase or the ALT mechanism. Here, aberrant cells may have gone through a phase of very short telomeres, but survived crisis and evolved to clones after telomere-elongating mechanisms were triggered. Correspondingly, patients with a less severe subtype and mild telomere shortening might not show significant differences compared to patients with advanced MDS, whose telomeres have probably at an earlier stage gone through a phase of excessive shortening, but have already been re-elongated.

Thus, it became clear that the different cohorts were too heterogeneous, as the disease status and the time point after diagnosis have to be taken into account. To gain more information, we subsequently performed follow-up analyses in patients with MDS with an isolated deletion of 5q [62]. At an early time point after initial diagnosis and prior to lenalidomide treatment, patients who had a later disease progression showed significantly shorter telomeres than patients without progression. At the time point of progression, all patients showed a re-elongation of the median telomere length that was similar to that measured in patients without progression. Thus, at an early time point after diagnosis, short telomere length seems to indicate an increased risk of relapse and disease progression, and with this provides further evidence of telomere shortening playing a fundamental role in the malignant transformation of hematopoietic stem cells. In general, the actual telomere length has to be interpreted carefully, depending on different factors such as disease state and treatment. In MDS, it is not yet clear whether telomere shortening is a causative factor, as in dyskeratosis congenita, or whether the increased proliferation of MDS cells concomitantly leads to telomere shortening. On the practical side, measurement of telomere length in patients with MDS and AML could therefore be a tool for selecting those patients who might, in the first step, be monitored more closely to recognize disease progression at an early stage. Thus, telomeres not only play a role in disease pathogenesis and progression, but they may also serve as an important tool for risk assessment and therapeutic decisions.

## 3. Epigenetics

### 3.1. Mutations of Epigenetic Regulators

Somatic mutations in epigenetic regulators are a common genetic event in myeloid neoplasia and contribute to hematopoietic transformation. Epigenetic regulators can change the gene expression without changing the nucleotide sequence. Genomic DNA is wrapped around the histone proteins (H2A, H2B, H3 and H4), whose N-termini can be modified, constituting the chromatin. These modifications include acetylation, methylation, ubiquitylation, phosphorylation and SUMOylation. A strong interaction between the genomic DNA and the histones results in a compact chromatin formation, whereas weak interactions enable gene expression by an open chromatin structure. Analyses of recurring translocations like t(11;16)(q23;p13.3), documented in cases of secondary AML or MDS, revealed the epigenetic translocation partners MLL (histone methyltransferase) and CBP (histone acetyltransferase) for the first time [63]. Up to now, many epigenetic modifiers are shown to be involved in translocations like *MOZ* [64], a histone lysine acetyltransferase or *NUP98*, which provide evidence of the importance of deregulated epigenetic factors.

Innovative technologies like array comparative genomic hybridization (arrayCGH), single nucleotide polymorphism (SNP) arrays and next-generation sequencing revealed recurrent mutations of genes implicated in epigenetic control such as *ASXL1*, *EZH2*, *DNMT3A* and *TET2* in MDS and AML.

The human *ASXL1* gene is located on chromosome 20q11.21 and codes for part of the Polycomb-repressive deubiquitinase complex, which functions to deubiquitylate H2AK119 [65]. *EZH2* located on chromosome 7q36.1, is a histone methyltransferase that catalyzes trimethylation of H3K27 and is a subunit of the polycomb repressive complex 2 (PCR2). Polycomb proteins initiate and maintain transcriptional silencing through specific histone modifications. DNMT3A is a member of the mammalian family of methyltransferases that enzymatically add a methyl group to cytosine in CpG dinucleotides (cytosine and guanine separated by only one phosphate). Methylation of CpG sites within the promoters can lead to gene silencing, a frequent mechanism to inactivate tumor suppressor genes. Examples in MDS are *KLF5*, *KLF11*, and *MAFB*, shown to be aberrantly hypermethylated in 15%, 7%, and 1.7% of 115 cases, respectively [66].

The Tet family was first identified as an oncofusion partner of the histone H3 Lys4 (H3K4) methyltransferase MLL in patients with the translocation t(10;11)(q22;q23) in AML [67]. Recent studies demonstrate that Tet proteins catalyze the conversion of 5-methylcytosine of DNA to 5-hydroxymethylcytosine (5hmC) and that *TET2* mutations are associated with low 5hmC levels and global hypomethylation [68]. This suggests that an altered 5hmC status leads to deregulation of important hematopoietic regulators and contributes to malignancy [69].

### 3.2. Role of Global DNA and Histone Methylation

Novel techniques based on mass spectrometry allow large-scale quantitative DNA methylation analysis [32]. Using this approach, Bullinger *et al*. were able to report the first large-scale methylation-based outcome predictor in AML. Furthermore, they observed distinct methylation patterns for cytogenetic subgroups such as AML with inv(16), t(8;21), t(15;17) and t(11q23). Also, microarray-based methods help to identify new clinical markers especially for AML with a normal karyotype and define new subgroups of AML. These techniques also allow the analysis of single promoter methylation patterns of specific genes. Promoter CpG methylation is often correlated with silencing of TSGs (tumor suppressor genes) in specific pathways that are also targets of mutation or other mechanisms of inactivation. Notably, TSGs silenced epigenetically often reside in genomic regions that are characterized by frequent chromosomal deletions. These results indicate that aberrant methylation can cooperate with chromosome deletions to silence TSG.

Using genome-wide approaches, unique methylation patterns of MDS and secondary AML were detected. Although particular cancer-related genes such as *CDKN2A* and genes in the WNT signaling pathway were hypermethylated, epigenetic deregulation was not limited to cancer-associated genes but appeared to be a more widespread phenomenon [70].

Comparing low-risk MDS cases with a control group, 552 differentially methylated CpG loci were identified, while hypermethylated genes were more frequent than hypomethylated genes [71]. In another study, patients with higher levels of methylation, compared with patients with lower levels, had a shorter median overall survival (12.3 *v* 17.5 months, respectively) and shorter median progression-free survival (6.4 *v* 14.9 months, respectively) [72]. Shen *et al*. used these methylation analyses as a prognostic model and observed that this was independent of age, sex, and IPSS group.

So far, it is a matter of discussion whether mutation of single epigenetic regulators induce genetic instability. EVI1, a hematopoietic transcription factor that regulates stem cell renewal and induces epigenetic modifications [73], is frequently overexpressed in human myeloid neoplasia [74]. *EVI1* rearrangement due to inv(3) or t(3;3) is frequently associated with monosomy 7 and a very poor prognosis [75]. *EVI1* is also a site of ectopic integration of retroviral vectors. Integration of a single retroviral vector into the *EVI1* locus has led to leukemogenesis in mice [76,77]. Notably, in a human gene therapy trial, insertional mutagenesis was accompanied by the development of monosomy 7. There are contradicting results as to whether EVI1 overexpression directly induces CIN via defective cytokinesis and accumulation of supernumerary centrosomes [78]. Thus, preleukemic dominance as a result of spontaneous chromosomal rearrangements or transgene integration events may be related to inhibited differentiation of hematopoietic stem cells [74].

Epigenetic modifications like DNA and histone methylation also play an important role in the induction of genomic instability. With the aim of studying the cooperation of genetic alterations and epigenetic modifications in the induction of genomic instability in leukemogenesis, we established a bone marrow transplantation model of myeloid leukemia in mice. The genetic defect resulted from an overexpression of the master oncogene *c-Myc*, whereas the epigenetic defect was induced by the histone methyltransferase *Suv39h1* deficiency. Suv39h1 mediates the trimethylation of lysine 9 of histone 3 (H3K9me3) leading to condensed chromatin.

As expected, clonal chromosomal aberrations induced by *c-Myc* overexpression were found in *c-Myc/*wt leukemias of about one-third of primary recipients and in more than 80% of secondary recipients [79]. Notably, leukemias that arose in *c-Myc/Suv39h1*-null mice were chromosomally stable, demonstrating that reduced H3K9 trimethylation due to lack of *Suv39h1* rather protects leukemic cells from the development of CIN.

In our further analyses, we identified centromeric fusions with loss of telomeric signals and higher numbers of critically short telomeres in *c-Myc/Suv39h1*-wt cells, but not in *Suv39h1*-null cells. Thus, telomere shortening to critical levels may have been prevented in *Suv39h1*-null cells, possibly due to better access of telomere-stabilizing proteins because of a more open chromatin structure. This is in accordance with the observation by Garcia-Cao *et al*. [80], who proved elongated telomeres in *Suv39h1* and *Suv39h2* double knock-out cells compared to wt cells. The critically short telomeres in leukemic *c-Myc/Suv39h1*-wt cells may impair chromosomal stability by inducing breakage-fusion-bridge cycles resulting in e.g., centromeric fusions [81]. Furthermore, missegregation driven by telomere shortening may cause numerical chromosomal aberrations [82]. In our BM transplantation model, numerical chromosomal aberrations were found in primary recipients and centromeric fusions were found mostly in secondary recipients. This may indicate that progressive telomere shortening may be involved in clonal evolution, *i.e*., “mild” telomere shortening inducing chromosomal missegregation and numerical aberrations and more pronounced telomere shortening resulting in centromeric fusions and complex chromosomal rearrangements.

The heterogeneous telomere lengths and the detection of ALT-associated PML bodies (APBs) indicated that the ALT mechanism was mainly responsible for telomere maintenance in *c-Myc*-induced leukemias. Microarray analyses identified an increased expression of genes involved in the ALT mechanism such as *Pml*, *Rad50*, *Smc5*, *Fen1*, *FancA*, *Mus81*, *Sp110* and *Sp100* in *c-Myc/Suv39h1*-null leukemias. This is in agreement with the observation that knock-down of *Rad50c* and *Smc5* inhibits ALT-induced telomere maintenance, resulting in critically short telomeres [83]. Downregulation of *Fen1*, *FancA* and *Mus81* prevents telomeric recombination required for ALT [84]. The more effective elongation or stabilization of telomeres in *c-Myc*-driven myeloid leukemias by ALT mechanism may prevent telomere shortening and the induction of CIN in *Suv39h1*-deficient leukemias.

Consistent with studies by Karlsson *et al*. [85] and Ray *et al*. [86], we demonstrated a *c-Myc*-induced increase of DNA double-strand breaks (DSBs). However, DSBs were markedly reduced in *c-Myc/Suv39h1*-null leukemias. Genes coding for repair proteins like *Rad51c*, *Trp53bp1* or *Ccnf* were upregulated in leukemic *c-Myc/Suv39h1*-null cells. It has been shown that reduced expression of *Rad51c*, *Trp53bp1* or *Ccnf* is linked to chromosomal aberrations like sister chromatid fusions, aneuploidy or tetraploidy [87,88,89]. The up-regulation of these genes in leukemic *c-Myc/Suv39h1*-null cells may strengthen genomic stability due to improved DSB repair. Moreover, changes in the chromatin structure in *Suv39h1*-null cells may improve the accessibility of the DNA repair machinery. As shown by Goodarzi *et al*. [90], heterochromatin can be a barrier for physiological DSB repair and *Suv39h1* and *Suv39h2* double knock-out diminish DSB repair defects following *Atm* knock-out. Thus, highly efficient DSB repair might be a potential mechanism for preventing chromosomal aberrations in *c-Myc/Suv39h1*-null leukemias. Our data showed for the first time that *Suv39h1* deficiency may prevent genomic instability by more efficient DNA repair and telomere stabilization in hematopoietic bone marrow cells overexpressing *c-Myc.*

## 4. Conclusions

Even though there are many hints, the exact mechanism of chromosomal instability is still unclear. As we discuss in this review, telomere shortening and epigenetic deregulation may be involved in the development of chromosomal and genomic instability. Importantly, in leukemia, genomic instability and in particular CIN may lead to the evolution of a complex aberrant karyotype, which is associated with a poor prognosis. This raises the question whether early intervention in aberrant epigenetic processes or telomeric dysregulation may prevent the outgrowth of aberrant clones. Telomere length measurement may be integrated in risk assessment to predict the prognosis. Thus, telomeres and epigenetics most likely play an important role in the induction of chromosomal instability in cancer and particularly in myeloid neoplasia. Elucidation of the exact mechanism of CIN induction in myeloid neoplasia remains a promising aim to understand leukemogenesis and find new therapeutic approaches.
